# Supplementary material for: Obesity prevention in the early years: A mapping study of national policies in England from a behavioural science perspective
Source: PLoS One. 2020 Sep 30;15(9):e0239402. doi: 10.1371/journal.pone.0239402 (PMC7526925; doi:10.1371/journal.pone.0239402)
Supplement: Attachment — Submitted filename: Response to Reviewers.docx [file pone.0239402.s002.docx]

Dr Joerg Heber

**LONDON’S GLOBAL UNIVERSITY**

Editor-in-Chief

PLOS ONE

18^th^ August 2020

Dear Editor,

Please find enclosed revision of a mapping study of national policies in England for your consideration. We thank the reviewers for the very positive review and helpful comments.

We outline below the amends made in response to the following comment:

The UK Government's new obesity strategy driven by the COVID-19 pandemic will be relevant to this review. A reference to this under discussion would add to this topic.

We have added reference to this strategy in the discussion (page 21):

*“The recently published policy paper ‘Tackling obesity: empowering adults and children to live healthier lives’ (40) was prompted by increasing evidence of a link between obesity and severity of COVID-19 symptoms (41). A combination of individual level approaches (a weight management campaign and expansion of NHS obesity services) and environmental measures (such as legislation for calorie labelling and greater advertising restrictions) are outlined (40).”*

We have also added this to the limitations (page 22):

“In addition, this is a fast moving field, the Childhood Obesity Plan (Chapter 3) was published as part of the Prevention Green Paper after the mapping work was completed and therefore not included (42), *neither was the most recent policy paper addressing obesity (40).”*

We hope that this addresses this comment adequately. Thank you for your consideration.

Yours faithfully,

Helen Croker RD PhD

On behalf of the authors
